# Thermal‐Mechanical Coupling Performance of Heat‐Resistant, High‐Strength and Printable Al‐Si Alloy Antisymmetric Lattice Structure

**DOI:** 10.1002/advs.202407107

**Published:** 2024-08-29

**Authors:** Jiaqi Yan, Zhicheng Dong, Ben Jia, Shunshun Zhu, Guowei Li, Yuhao Zheng, Heyuan Huang

**Affiliations:** ^1^ School of Aeronautics Northwestern Polytechnical University Xi'an 710072 China; ^2^ School of Civil Aviation Northwestern Polytechnical University Xi'an 710072 China; ^3^ National Key Laboratory of Aircraft Configuration Design Xi'an 710072 China

**Keywords:** 3D printing, Al‐Si alloy, thermal‐mechanical coupling, lattice structures design, experiments and numerical simulations

## Abstract

The unsatisfactory mechanical performance at high temperatures limits the broad application of 3D‐printed aluminum alloy structures in extreme environments. This study investigates the mechanical behavior of 4 different lattice cell structures in high‐temperature environments using AlSi12Fe2.5Ni3Mn4, a newly developed, heat‐resistant, high‐strength, and printable alloy. A novel Antisymmetric anti‐Buckling Lattice Cell (ASLC‐B) based on a unique rotation reflection multistage design is developed. Micro‐CT (Computed Tomography) and SEM (Scanning Electron Microscope) analyses revealed a smooth surface and dense interior with an average porosity of less than 0.454%. Quasi‐static compression tests at 25, 100, and 200 °C showed that ASLC‐B outperformed the other 3 lattice types in load‐bearing capacity, energy absorption, and heat transfer efficiency. Specifically, the ASLC‐B demonstrated a 51.56% and 44.14% increase in compression load‐bearing capacity at 100 and 200 °C compared to ASLC‐B(AlSi10Mg), highlighting its excellent high‐temperature mechanical properties. A numerical model based on the Johnson‐Cook constitutive relationship revealed the damage failure mechanisms, showing ASLC‐B's effectiveness in preventing buckling, enhancing load‐transfer efficiency, and reducing stress concentrations. This study emphasizes the importance of improving energy absorption and mechanical performance for structural optimization in extreme conditions. The ASLC‐B design offers significant advancements in maintaining structural integrity and performance under high temperatures.

## Graphical abstract

A newly developed antisymmetric pyramid lattice cell is fabricated by SLM (Selective Laser Melting), and microscopic characterization is conducted using Micro‐CT (Computed Tomography) and SEM (Scanning Electron Microscope). The newAlSi12Fe2.5Ni3Mn4 alloy exhibits excellent properties in high‐temperature environments. Mechanical properties of the structures are obtained by experiments and numerical simulations under various temperatures. This article is protected by copyright. All rights reserved.

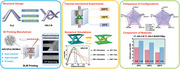

## 1 Introduction

Hypersonic vehicles encounter significant mechanical and aerodynamic thermal loads during flight, necessitating strict requirements for thermal protection systems and structural design.^[^ ^1^, ^2^, ^3^ ^]^ Existing thermal protection technologies can safeguard high‐temperature areas like the leading edge of fuselages but still face challenges when applied to sub‐high‐temperature zones like the leeward side, which ranges from 150 to 200 °C, posing serious concerns such as structural inefficiencies and excessive manufacturing costs.^[^ ^4^, ^5^, ^6^ ^]^ The integrated design of lattice structure presents dual advantages in structural load‐bearing and heat dissipation, demonstrating a promising direction for the development of ‘heat‐resistant and load‐bearing integrated’ structures.^[^ ^7^, ^8^, ^9^ ^]^

Aluminum alloy is increasingly popular in the designs of lattice‐structure thermal protection due to its lightweight property and great 3D printing efficiency.^[^ ^10^, ^11^ ^]^ High‐temperature‐resistant materials, such as titanium alloys,^[^ ^12^, ^13^, ^14^ ^]^ nickel alloys,^[^ ^15^, ^16^, ^17^ ^]^ and C/SiC ceramic matrix composites,^[^ ^18^, ^19^, ^20^ ^]^ are commonly employed in hypersonic vehicles. However, their complex preparation processes and suitability only for operational temperatures above 1500 °C limit their application. In contrast, high‐temperature‐resistant aluminum alloy offers a better solution for achieving lightweight and cost‐effective structures operating at environmental temperatures between 150 to 200 °C. Although the current manufacturing process can generally preserve the relatively good mechanical performance and load‐bearing capacity of aluminum alloy aluminum within this temperature range, it often fails to meet the accuracy requirements for processing complex structures.^[^ ^21^ ^]^ In comparison, additive manufacturing technology offers greater design flexibility and processing accuracy, with which reaching ±0.1 mm, making it suitable for manufacturing structures with intricate configurations.^[^ ^22^, ^23^, ^24^ ^]^ However, current aluminum alloy powders used for 3D printing, such as AlSi10Mg, lose over 20% of their strength when exposed to temperatures above 200 °C.^[^ ^25^ ^]^ Researchers have discovered that adding appropriate elements such as Fe, Ni, and Mn during the manufacturing process leads to the precipitation of significant quantities of Al5(FeNi) and Al6Mn phases, thereby improving the high‐temperature resistance and thermal stability of the Al‐Si alloy.^[^ ^26^, ^27^, ^28^ ^]^ However, little research has been done on printable high‐temperature resistant aluminum alloys. Therefore, developing printable high‐temperature‐resistant aluminum alloys is crucial for enhancing the thermal‐mechanical coupling performance of multifunctional lattice structures.

In engineering structural design, geometric design methods inspired by letter shapes have garnered widespread attention due to their symmetry and unique mechanical properties.^[^ ^29^, ^30^, ^31^ ^]^ The horizontal beam in an A‐shaped structure can effectively suppress buckling of the inclined side supports, significantly improving the load‐bearing characteristics with a minimal increase in mass. On the other hand, an antisymmetric multipolar design can create a stress equilibrium zone in the central part of the structure, effectively reducing local stress concentrations and enhancing the overall load‐bearing capacity.^[^ ^32^, ^33^ ^]^ Therefore, inspired by the mechanical principles of the A‐shaped structure and the geometric antisymmetric multipolar concept, the design of lattice structures with significant load‐bearing capacity and buckling resistance demonstrates distinctive innovation. Combining geometric optimization with mechanical analysis to achieve comprehensive improvements in structural performance is crucial for advancing the development of high‐performance lattice structures. In the design of innovative lattice structures, researchers have carried out various improvements and optimizations of lattice cell configurations by applying multistage structural design approaches.^[^ ^34^, ^35^, ^36^, ^37^ ^]^ Bhat et al. proposed a novel sea urchin (SU) lattice cell structure and conducted a nested multistage design.^[^ ^38^ ^]^ Experimental results showed that compared to the original structure, embedding an octahedral multistage structure within the SU increased the elastic modulus and compressive strength by 35.5% and 6%, respectively. Xiao et al. conducted a hybrid multistage design combining rhombic dodecahedron (RD) and octet lattice cells.^[^ ^39^ ^]^ Experimental and numerical analyses showed that the specific energy absorption of the hybrid lattice structure increased by 164.3% compared to the RD lattice structure and by 57.5% compared to the octet lattice structure. Wang et al. found that single‐stage TC4 (Ti‐6Al‐4V) pyramid lattice cells predominantly fail due to buckling and thus incorporated a secondary core design to reinforce the buckling‐prone location,^[^ ^40^ ^]^ which the optimized multistage structure improved load‐bearing capacity by 18.4% at 25 °C and 23.0% at 350 °C, respectively. Moreover, multistage lattice structures can improve convective heat transfer by increasing the heat exchange area, which optimizes the active cooling thermal protection for the structure.^[^ ^41^, ^42^, ^43^, ^44^, ^45^ ^]^ Hence, the objective of this study is to employ the multistage structural design approach in order to establish a newly developed design methodology for lattice structures made of high‐temperature‐resistant aluminum alloys.

This study investigated the load‐bearing characteristics and failure mechanisms of 4 lattice cell structures manufactured by the heat‐resistant and high‐strength AlSi12Fe2.5Ni3Mn4 alloy powder in high‐temperature environments. First, a newly designed Antisymmetric anti‐Buckling Lattice Cell (ASLC‐B), designed based on the concept of rotation reflection multistage principles, was fabricated via Selective Laser Melting (SLM), alongside 3 control cells (PLC (Pyramid Lattice Cell), PLC‐B (Pyramid anti‐Buckling Lattice Cell), and ASLC (Antisymmetric Lattice Cell)). The print quality of these specimens was confirmed through characterization methods such as Scanning ElectronMicroscope (SEM) and 3D Micro‐CT (Computed Tomography), ensuring suitability for subsequent testing. Second, various experiments were conducted to determine the basic mechanical properties of the raw alloys, Johnson‐Cook (J‐C) constitutive parameters, and the load‐bearing capacity as well as failure modes of the 4 AlSi12Fe2.5Ni3Mn4 lattice cell structures at 25, 100, and 200 °C, respectively. Meanwhile, the load‐bearing characteristics, energy absorption effect, and heat‐transfer efficiency of lattice cells with different configurations and materials (AlSi10Mg) were compared within these 3 temperatures. Finally, the high‐precision finite element model based on the J‐C constitutive relationship was used to uncover the deformation failure processes of the 4 configurations across different temperatures. This study conveyed that optimizing the structures and improving the material mechanical properties provided valuable engineering guidance to near‐space hypersonic vehicles under extreme conditions.

## 2 Results

### 2.1 Structural Design

**Figure 1:**
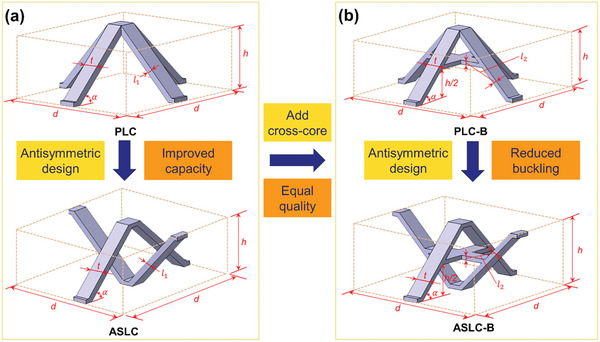
Designing concept of lattice cell: a) antisymmetric method; b) anti‐buckling method.

It has been confirmed that the PLC structure was highly flexible in design and provided remarkably efficient heat dissipation.^[^ ^46^, ^47^, ^48^ ^]^ In this study, we adopted a multistage rotation reflection design method based on the pyramid lattice cell and the anti‐Kagome lattice structure proposed by Han.^[^ ^49^ ^]^ By using a one‐rod core of the PLC as the basis and performing a 180° rotation reflection within the plane of the structure's spatial center, a newly designed ASLC was obtained, as shown in **Figure** **1**a. The resistance to buckling of the rod core significantly influenced the load‐bearing capacity of the overall lattice structure, by which the core at the buckling point of the pyramid lattice structure needs to be reinforced. Consequently, a secondary cross‐core has been incorporated into the rod core for anti‐buckling constraint, resulting in a PLC‐B and an ASLC‐B. The cross‐core design reduced the length of the single‐core, effectively preventing initial buckling damage, as shown in the specific design concept in Figure 1b.

Figure 1 shows the structural parameters of the 4 lattice cell types, where *d *= 30 mm, *h *= 16 mm, *t *= 4 mm, and *α *= 45°. To ensure that the relative density of the 4 lattice cells remained unchanged, the core thickness of PLC and ASLC was set as *l_1 _* = 1.5 mm, while the core thickness of PLC‐B and ASLC‐B was set as *l_2_* = 1.15 mm.

### 2.2 Preparation and Testing

AlSi12Fe2.5Ni3Mn4 is a high‐temperature‐resistant aluminum alloy powder designed suitably for selective laser melting (SLM) 3D printing. Developed and provided by Jiangxi Baohang Advanced Materials Co., this material is based on Al‐Si alloy, with composite additives that improve high‐temperature creep resistance, solving the problem of inadequate high‐temperature performance found in traditional aluminum alloy printing materials (www.bhmaterials.cn). Also, test pieces used in this study are provided by Jiangxi Baohang Advanced Materials Co., Ltd.

The test pieces fabricated using SLM technology include basic mechanical performance test pieces and lattice structure test pieces. As shown in Figure S1a,b (Supporting Information), SEM images of AlSi12Fe2.5Ni3Mn4 and AlSi10Mg powders prepared by gas atomization process indicated that the particle size ranges from 10–35 µm. And the densities of AlSi12Fe2.5Ni3Mn4 and AlSi10Mg alloys were measured to be 2.70 and 2.68 g cm^−3^, respectively.

The laser source power for printing was 370W, with a spot diameter of 0.07 mm, a scanning speed of 1200 mm s^−1^, a hatch distance of 0.2 mm, a layer distance of 0.03 mm, a region overlap set to 0.1 mm, a region width set to 12 mm, and scanning direction in the opposite direction of the airflow. Figure S1c (Supporting Information) shows a schematic diagram of the SLM production process. The basic mechanical performance test pieces consisted of standard tensile, compression, shear, and thermal expansion test specimens; the lattice cell test pieces included PLC, PLC‐B, ASLC, and ASLC‐B designs.

The results of the basic mechanical performance tests are presented in **Table** **1**. In addition, as shown in Figure S2 (Supporting Information), tensile tests for AlSi10Mg were conducted at 25, 100, and 200, and the test results were consistent with the reference.^[^ ^53^ ^]^

**Table 1:** Basic mechanical performances of AlSi12Fe2.5Ni3Mn4.

Temperature [°C]	*E* [GPa]	*µ*	*σ_b_* [MPa]	Elongation at fracture [%]	*σ_c_* [MPa]	*τ* [MPa]	*a* [E‐6·1/K]
25	69.00	0.33	554.87	5.32	734.38	238.06	–
100	64.50	0.33	519.65	9.19	602.58	191.09	20.58
200	53.00	0.33	372.04	14.36	523.75	161.94	25.13

^a)^ *E* and *µ* are the Elastic modulus and Poisson's ratio;

^b)^ *σ_b_*, *σ_c_*, *τ* are the nominal tensile ultimate strength, nominal compressive ultimate strength, and nominal shear ultimate strength;

^c)^ *a* is the thermal expansion coefficient.

**Figure 2:**
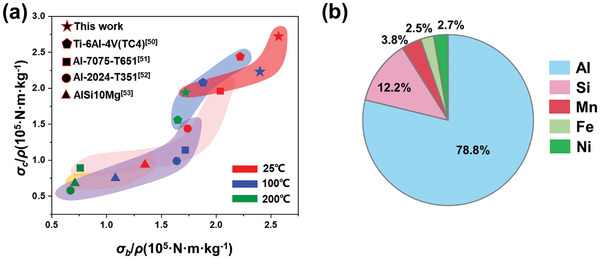
a) Comparison of mechanical performances of AlSi12Fe2.5Ni3Mn4 with common metal materials^[^ ^50^, ^51^, ^52^, ^53^ ^]^; b) Elemental analysis results of AlSi12Fe2.5Ni3Mn4 by EDS.

**Figure** **2**a displayed a comparison of the specific tensile strength and specific compressive strength of AlSi12Fe2.5Ni3Mn4 against other commonly used metal materials. It was evident that in a room‐temperature environment, the specific tensile strength of AlSi12Fe2.5Ni3Mn4 surpassed that of AlSi10Mg by 52.59% and Al‐2024‐T351 by 18.39%. Remarkably, it also surpassed the specific tensile strength of Al‐7075‐T651 by 81.58% and achieved performance comparable to TC4 titanium alloy in high‐temperature environments. Additionally, to provide a detailed insight into the composition of AlSi12Fe2.5Ni3Mn4 alloy, an elemental analysis was conducted using Energy Dispersive Spectroscopy (EDS) and X‐ray Diffraction (XRD), with the result displayed in Figure 2b and Figure S1f,g (Supporting Information). The above results provide sufficient evidence of the constituent elements of the alloy.

30 × 30 × 1 mm^3^ upper and lower panels were employed to ensure uniform load distribution during the test. The compression test results of the lattice structures are shown in **Table** **2**. The comparison of these results reveals that the ASLC‐B printed with AlSi12Fe2.5Ni3Mn4 exhibited the highest load‐bearing capacity at different temperatures.

**Table 2:** Average ultimate load (kN) of 4 configurations of lattice cells.

Configurations	25 °C	25 °C‐AlSi10Mg	100 °C	100 °C‐AlSi10Mg	200 °C	200 °C‐AlSi10Mg
PLC	5.43	4.20	4.55	3.55	3.87	2.82
PLC‐B	6.83	5.26	5.52	4.38	4.86	3.59
ASLC	5.40	4.19	4.57	3.54	3.79	2.82
ASLC‐B	7.35	5.31	6.13	4.47	5.01	3.65

^a)^ 25/100/200 °C‐AlSi10Mg represent the test results of specimens printed with AlSi10Mg at temperatures of 25/100/200 °C.

### 2.3 Numerical Simulation

The J‐C constitutive parameters of newly developed AlSi12Fe2.5Ni3Mn4 aluminum alloy obtained from tests and least squares fitting are shown in **Table** **3**.

**Table 3:** J‐C constitutive parameters of AlSi12Fe2.5Ni3Mn4.

*A*	*B*	*C*	*m*	*n*	*T_r_*	*T_m_*
551.72	735.93	0.0174	1.68	0.64	25	790

**Figure 3:**
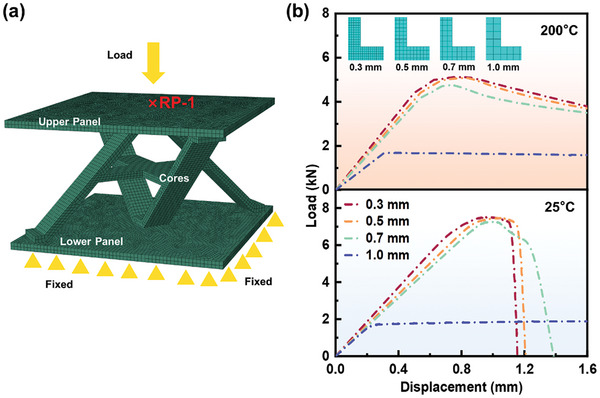
a) The finite element model of ASLC‐B; b) Convergence results of ASLC‐B with different mesh sizes (25 and 200 °C).

To verify the convergence of the finite element model, the ASLC‐B at 25 and 200 °C was analyzed using different mesh sizes. **Figure** **3**b illustrates the mesh distribution of the 1/4 cross‐core with different mesh sizes. The results showed that at a mesh size of 1 mm, there is a significant discrepancy between the numerical simulation and the actual result, indicating that the model does not converge. When the mesh size was reduced to 0.7 mm, the accuracy of the simulation results improved noticeably. Further refinement of the mesh to 0.5 mm and then to 0.3 mm resulted in numerical simulations that increasingly align with the actual situation, demonstrating convergence and increasing the reliability of the model. Therefore, a 0.5 mm finite element mesh size had high computational efficiency, providing convergent calculation results that were consistent with the experiments and could more accurately simulate the compression deformation process of the lattice structure across different environmental temperatures.

## 3 Discussion

### 3.1 Microscopic Characterization of Test Specimens

**Figure 4:**
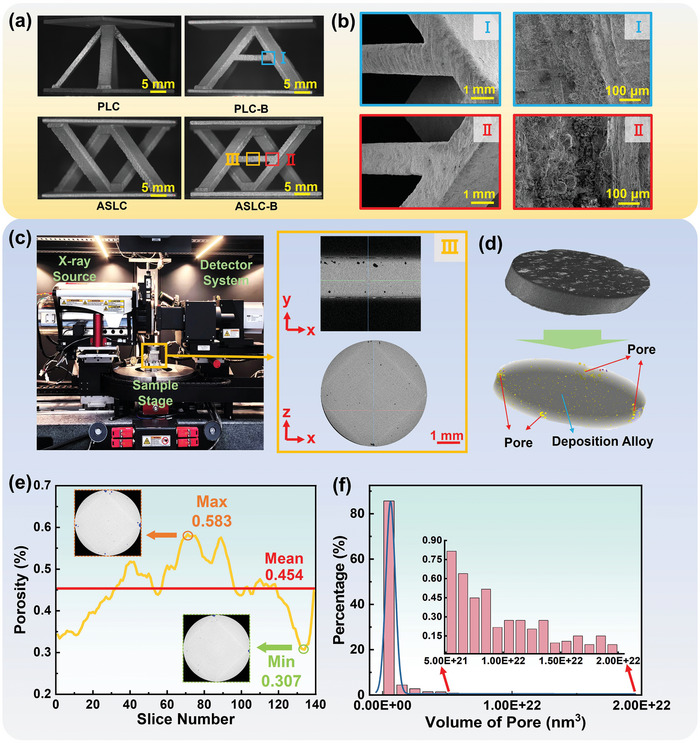
a) Physical printing images of lattice cells; b) SEM images of PLC‐B and ASLC‐B at the core connection; c) Micro‐CT device and the schematic of scanning results; d) Distribution and 3D morphology of pores; e) Porosity distribution curve in the direction of deposition; f) Frequency distribution of pore volume.

**Figure** **4**a displays the physical printing image of 4 lattice cell configurations: PLC, PLC‐B, ASLC, and ASLC‐B. Figure 4b shows the surface morphology of the core connection of PLC‐B and ASLC‐B, obtained using a 3D digital microscope equipped with a super depth of field video (model: Smartzoom5) at different SEM magnifications. The microscopy results indicated that the surface morphology of the lattice cell specimens prepared using AlSi12Fe2.5Ni3Mn4 was smooth, indicating excellent print formability at the core connection area. The deposited powder was densely packed and retained high sphericity. In addition, the laser metal deposition structure of the entire AlSi12Fe2.5Ni3Mn4 exhibited continuous layering, indicating that the printing quality of specimens meets the necessary requirements for structural integrity and performance.

The porosity of the printed lattice structures was analyzed using CT technology. For this purpose, an X‐ray Micro‐CT system (model: ZEISS Xradia 620 Versa) equipped with a 120kV voltage and 25W output power was employed to inspect the AlSi12Fe2.5Ni3Mn4 lattice structure. Figure 4c displays the setup of the scanning device and the images of the scanning results obtained from different directions. By applying a global grayscale threshold to the volume model reconstructed by Micro‐CT, the segmentation of pores and deposited alloy within the test pieces was completed, allowing for the detailed determination of the pore distribution and morphology, as depicted in Figure 4d. It was clear that larger‐sized pores tended to be concentrated around the model, whereas smaller‐sized pores were evenly distributed throughout the interior of the reconstructed model.

Figure 4e displayed the specific pore morphology and overall porosity of the test piece, analyzed on a plane parallel to the deposition direction. The directional analysis has revealed a porosity gradient throughout the reconstructed model. The upper and lower regions exhibited low porosity, while the middle region showed high porosity. The porosity values ranged from as low as 0.307% to as high as 0.583%. After fitting and calculating the curve, the average porosity of the test piece was determined to be 0.454%. Moreover, Figure 4f presented the frequency distribution of pore volume within the test piece, where over 80% of the pore volume measured less than 10^20^ nm^3^. This distribution was further analyzed by integrating the SEM images and the 3D reconstructed model, as shown in Figure 4b,d, which helped to correlate the visual morphology with the quantitative porosity data.

### 3.2 Numerical Method Validation

**Figure 5:**
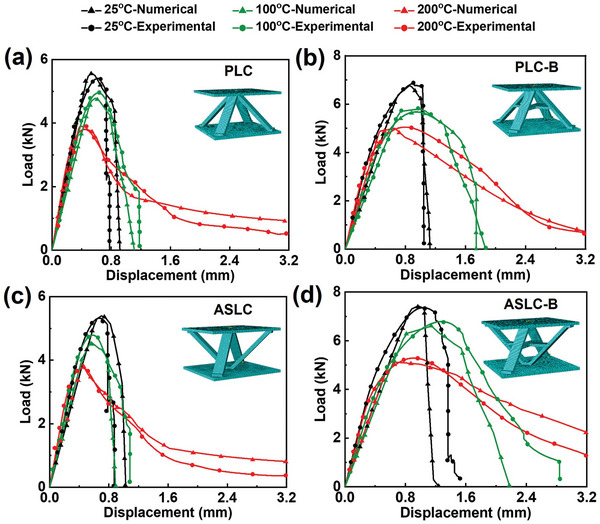
Experimental and numerical load‐displacement curves of AlSi12Fe2.5Ni3Mn4 lattice cells at 25, 100, and 200 °C: a) PLC, b) PLC‐B, c) ASLC, and d) ASLC‐B.

**Figure 6:**
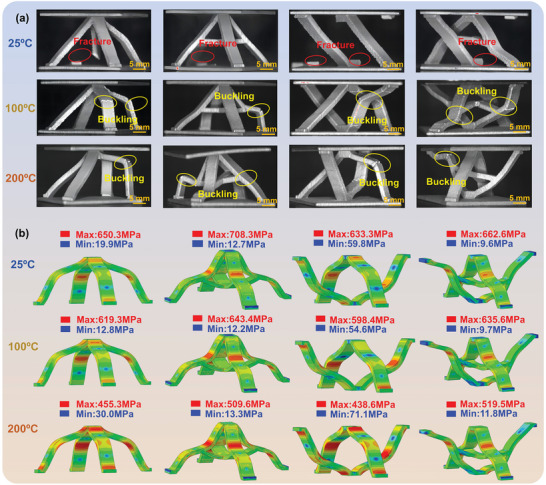
a) Physical failure images of 4 configurations; b) Von Mises stress distribution under failure state.

To verify the accuracy of the numerical model, **Figure** **5** displayed the load‐displacement curves for 4 configurations derived from both experiments and numerical simulations at 25, 100, and 200 °C. The experimental curves presented were those closest to the average ultimate load, ensuring a representative analysis. Physical failure images and failure stress clouds of 4 configurations at 25, 100, and 200 °C are shown in **Figure** **6**a,b, respectively. From Figure 5, it was evident that the trends in the load‐displacement curves from the numerical models align closely with those from the experimental curves across different temperatures. The maximum calculation error noted was only 4.59%, which was considered reasonable and within acceptable limits for such simulations, confirming the reliability of the numerical model and providing a solid foundation for further studies aimed at elucidating the damage evolution and failure mechanisms in lattice structures under various load and temperature.

According to the failure modes observed in Figure 6a, the 4 lattice structures exhibit no distinct plastic stage at 25 °C. Upon reaching the ultimate load, an abrupt decline occurred, with the structural failure mode characterized by the instantaneous fracture of cores. In high‐temperature conditions, the aluminum alloy tended to soften, reducing the structural load‐bearing capacity but enhancing ductility. Therefore, compared to the room‐temperature environment, the sudden drop in the load‐displacement curves at 100 and 200 °C was noticeably mitigated, and the duration of the plastic plateau extended. The structural failure mode under these conditions was predominantly characterized by the core buckling. By analyzing the failure stress clouds of numerical simulations, as shown in Figure 6b, the stress‐concentrating in the 4 lattice structures consistently appeared at the junctions between cores and panels, on the inner side of the direction of cores buckling, and at the cross‐cores. Compared with Figure 6a, these stress‐concentrating areas in the numerical simulation aligned well with the locations of instantaneous core fractures and fractures caused by buckling deformation, further validating the effectiveness of numerical simulations in predicting the actual behaviors of the structures.

Upon analyzing the stress distribution in 4 lattice structures at 25 °C, it was obvious that the inclusion of cross‐cores in the PLC‐B and ASLC‐B effectively restrained the initial buckling, thus reducing stress‐concentrating areas compared to the PLC and ASLC. By dispersing large‐scale buckling into small‐scale buckling in multiple directions, this design strategy may mitigate these unsatisfactory buckling and enhance structural stability. On the other hand, comparing antisymmetric structures to pyramid structures, it appeared that reversing the structure scattered the contact points between the core and panel. This scattering led to uneven distribution of the load‐bearing capacity across the core, resulting in the appearance of asymmetric buckling. This phenomenon was particularly obvious in ASLC‐B, where unilateral stress‐concentrating occurred at the connection between the core and the lower panel, as validated by the experimental results shown in Figure 6a. Further examination of the stress distribution clouds at different temperatures in Figure 6b revealed that the overall stress distribution across the 4 lattice cell configurations remained consistent regardless of temperature increase. However, at high temperatures, buckling intensity escalated, and the severe coarsening of nano‐deposited phase particles in the aluminum alloy led to an expanded stress concentration area and reduced structural stress. Notably, at 200 °C, the structural stress of ASLC‐B decreased by 21.60% compared to that at 25 °C, marking the lowest reduction among the 4 configurations. These findings from the numerical simulations proved that the design of ASLC‐B, being particularly effective in high‐temperature environments, not only optimized the stress distribution of cores but also maintained the great load‐bearing capacity.

### 3.3 Effect of Configurations

**Figure 7:**
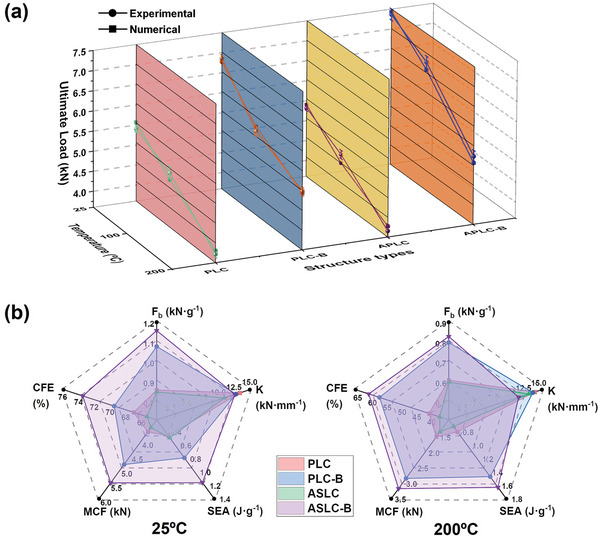
a) Experimental and numerical ultimate loads of PLC, PLC‐B, ASLC, and ASLC‐B at 25, 100, and 200 °C; b) Comparison of F_b_, SEA, MCF, CFE, and K for PLC, PLC‐B, ASLC, and ASLC‐B at 25 and 200 °C.

**Figure** **7**a illustrated the ultimate loads of 4 lattice cell structures obtained from experiments and numerical simulations at different temperatures. The error bars represented the variability of each group. Figure 7b offered a comprehensive comparison of the load‐bearing efficiency of the 4 lattice cells at 25 and 200 °C. The comparison leveraged quantified indicators of structural performance, including the load‐to‐weight ratio (F_b_), specific energy absorption (SEA), mean crushing force (MCF), crushing force efficiency (CFE), and structural stiffness (K).^[^ ^54^, ^55^, ^56^ ^]^

F_b_ represents the load‐bearing capacity per unit mass, with higher values indicating greater load‐bearing, which can be calculated as follows:

(1) $$F_{b}=\frac{F_{\max}}{M}$$

Here, *F_max_* is the structural ultimate load, and *M* is the total mass.

SEA measures the energy absorbed per unit mass. A higher SEA value indicates a greater capacity for energy absorption, with the formula as follows:

(2) $$\mathrm{SEA}=\frac{\int_{0}^{S}F\left(x\right)\mathrm{dx}}{M}$$

Here, *F(x)* is the compressive load and *S* is the failure displacement. In this study, *S* corresponds to the point of sudden drop in the load‐displacement curve. The structure is considered to have failed when it reaches a strain of 20% (Displacement = 3.2 mm) in high‐temperature environments, with the corresponding displacement point marked as *S*.

MCF represents the average load during the structural compression process, considering the load‐bearing capacity throughout both the plastic stage and the failure stage. The method for calculating MCF is as follows:

(3) $$\mathrm{MCF}=\frac{\int_{0}^{S}F\left(x\right)\mathrm{dx}}{S}$$

CFE represents the energy absorption efficiency. The higher CFE is, the greater the energy absorption efficiency can be. The calculation method of CFE is as follows:

(4) $$\mathrm{CFE}=\frac{\mathrm{MCF}}{F_{\max}}\;\times \;100\%$$

K represents the ability to resist deformation, characterized by the slope of the load‐displacement curve during the elastic stage.

First, the analysis of F_b_, mainly focusing on the ultimate loads of the 4 configurations, has been conducted. As shown in Figure 7a, the ultimate loads of PLC‐B and ASLC‐B at 25 °C were 6.86kN and 7.36kN, denoting the load‐bearing capacities have increased by 26.80% and 38.35%, compared to PLC and ASLC, respectively. The improvements indicated that the cross‐core design effectively constrained the initial deformation damage. By reducing the length of the single core, this design strategy enhanced local superstability and resistance to instability. On the other hand, the symmetry of the anti‐type design greatly affected force transmission. Analysis showed that the force direction of the core changed, with the core dispersed at the contact points with the panels, concentrating the main load‐bearing parts at the midpoint. Due to the lack of constraints, the load distribution became uneven, resulting in a slight decrease in the ultimate load of ASLC compared to PLC. According to the failure results at 200 °C in Figure 6a, ASLC exhibited multi‐point buckling compared to PLC. ASLC‐B, which integrated both the anti‐type structural design and cross‐core constraints, optimized the force‐transmission pathway more effectively than PLC‐B. Within the temperature range studied in this paper, this integration facilitated the maximum increase in ultimate load of up to 19.54%.

As the temperature increased to 100 and 200 °C, the aluminum alloy softened, causing a significant decrease in the ultimate load across all 4 configurations compared to room temperature. The maximum load decrease ratios at 100 and 200 °C reached up to 17.20% and 29.57%, respectively. Among these, ASLC‐B exhibited a relatively small decrease in ultimate load, with reductions of 7.74% at 100 °C—the least among the configurations, and 28.13% at 200 °C, which is also below the maximum decrease observed. According to Figure 7a, at 200 °C, ASLC‐B possessed increased ultimate load compared to PLC, PLC‐B, and ASLC by 38.85%, 4.75%, and 41.07%, respectively. At 25 and 100 °C, the increase ratios were 36.04% and 37.17% compared to PLC, showing that the load enhancement became more pronounced as the temperature rose. Therefore, the design of ASLC‐B effectively weakened the adverse impact of high‐temperature environments on material properties, resulting in an improved and superior load‐bearing capacity of ASLC‐B compared to the other 3 structures under both room‐temperature and high‐temperature environments.

Next, the energy absorption capacity was compared. As shown in Figure 7b, the SEA of ASLC‐B at 25 °C was 1.14 J g^−1^, which was notably higher than that of the other 3 structures. Due to the increased load‐bearing capacity, the peak of the load‐displacement curve was elevated, consequently resulting in a synchronous improvement in the energy absorption capacity. At 200 °C, the SEA of ASLC‐B was 1.61 J g^−1^, marking a substantial improvement of 41.23% compared to its performance at room temperature. Combined with the observations from Figures 5d and 6a, which suggest that, unlike the instantaneous fracture of cores at room temperature, the high‐temperature environment prolonged the plastic plateau phase of structures, thereby allowing for better energy absorption capacity. In addition, comparing the load‐displacement curves of 4 configurations at 200 °C in Figure 5, ASLC‐B not only delayed the buckling failure compared to PLC and ASLC but also surpassed PLC‐B in terms of peak load. Therefore, ASLC‐B has a higher SEA at 200 °C compared to the other 3 structures.

Figure 7b also displayed the MCF and CFE of PLC, PLC‐B, ASLC, and ASLC‐B at 25 and 200 °C. The data revealed that the load capacity of the structures was higher at 25 °C, and the failure displacement was shortened due to the instantaneous fracture of cores. Universally, both the MCF and CFE of all 4 structures were higher at room temperature compared to high temperatures, indicating more efficient energy absorption. At 200 °C, ASLC‐B possessed the highest MCF and CFE among the 4 structures, highlighting its capability to maintain a relatively high load capacity and energy absorption efficiency throughout the plastic and failure stages. Additionally, due to the design of cross‐cores, PLC‐B and ASLC‐B show smaller reductions in MCF and CFE from 25 to 200 °C compared to PLC and ASLC, effectively ensuring sustained load capacity and energy absorption efficiency even under high‐temperature conditions.

Finally, the analysis focused on the structural stiffness, which was mainly related to the thickness of the core. To maintain the relative density of the structures constant, the core thickness of PLC‐B and ASLC‐B was reduced by 23.33% compared to PLC and ASLC. However, this reduction resulted in only a 12.59% decrease in stiffness. Nevertheless, the decrease in stiffness shifted the structures from simply ‘brittle and hard’ to relatively ‘soft and ductile’, which was more conducive for energy absorption, as reflected in several other performance indicators.

To summarize, the design of ASLC‐B significantly enhanced the structural load‐bearing capacity both in room‐temperature and high‐temperature environments. Moreover, as illustrated in Figure 7b, ASLC‐B demonstrated superior mechanical performance across various environmental temperatures.

### 3.4 Effect of Materials

**Figure 8:**
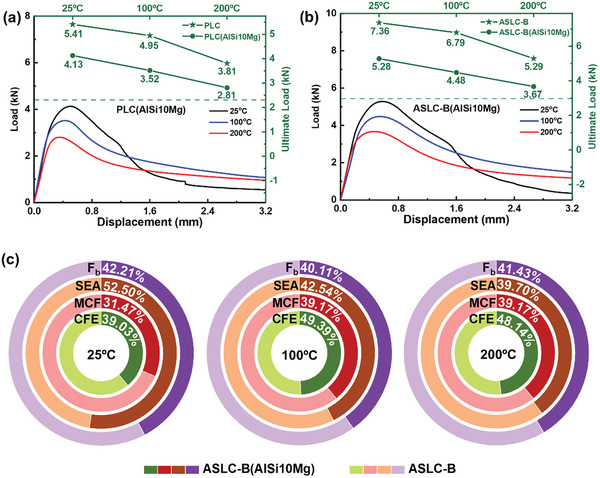
a) Load‐displacement curve of PLC(AlSi10Mg) and comparison with the ultimate load of PLC; b) Load‐displacement curve of ASLC‐B(AlSi10Mg) and comparison with the ultimate load of ASLC‐B; c) Comparison of F_b_, SEA, MCF, and CFE for ASLC‐B(AlSi10Mg) and ASLC‐B at 25, 100, and 200 °C.

To delve deeper into the effects of different materials, **Figure** **8**a,b presented the load‐displacement curves of PLC(AlSi10Mg) and ASLC‐B(AlSi10Mg) at different temperatures, alongside a comparison of ultimate loads between structures fabricated by AlSi10Mg and those by AlSi12Fe2.5Ni3Mn4. Figure 8c provided the normalized comparison of F_b_, SEA, MCF, and CFE for ASLC‐B(AlSi10Mg) and ASLC‐B at various temperatures, with values indicating the percentage contribution of ASLC‐B(AlSi10Mg) in relation to the sum of ASLC‐B(AlSi10Mg) and ASLC‐B.

**Figure 9:**
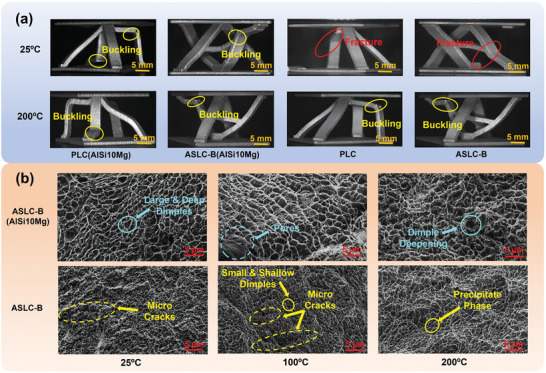
a) Failure schematics of PLC(AlSi10Mg), ASLC‐B(AlSi10Mg), PLC, and ASLC‐B at 25 and 200 °C; b) SEM images of fracture for ASLC‐B(AlSi10Mg) and ASLC‐B at 25, 100, and 200 °C.

As shown in Figure 5 in Section 3.2 and Figure 8, when examining the initial loading stage in the load‐displacement curves of structures printed by 2 materials, the curves were almost similar until reaching the ultimate load. At 25 °C, the AlSi10Mg lattice structures did not exhibit sudden drops in curves after reaching the ultimate load like AlSi12Fe2.5Ni3Mn4 ones. Instead, they underwent a small range of buckling deformation that gradually increased with continued loading, leading to significant buckling damage and a substantial decrease in the load‐bearing capacity. As shown in Figure 8a,b the ultimate loads of PLC(AlSi10Mg) and ASLC‐B(AlSi10Mg) at 25 °C were 4.13kN and 5.28kN. The load‐bearing capacity of PLC and ASLC‐B made of AlSi12Fe2.5Ni3Mn4 increased by 30.99% and 39.39%, respectively, when compared to their counterparts made of AlSi10Mg. Furthermore, the failure results of structures made from both materials at 25 °C, as shown in **Figure** **9**a, revealed that although the AlSi12Fe2.5Ni3Mn4 structures possessed a higher load‐bearing capacity, they exhibited reduced structural ductility and a shortened plastic plateau period.

At 100 and 200 °C, the load‐bearing capacity of ASLC‐B (as shown in Figure 8b) increased by 51.56% and 44.14%, respectively, compared to ASLC‐B(AlSi10Mg). Also, ASLC‐B exhibited a smaller decrease in ultimate load from room temperature to high temperatures, indicating its superior thermal stability. AlSi10Mg, while being more ductile at higher temperatures, was susceptible to oxidation and exhibited a relatively high thermal expansion coefficient in high‐temperature environments, which introduced significant thermal stresses in environments with large temperature differences. This may adversely affect the stability and internal quality of the material. Upon analyzing the structural failure morphology, it was evident that the AlSi10Mg structures demonstrated a higher level of material ductility in high‐temperature environments, as they did not display large‐scale structural fracture at the core buckling location. Furthermore, a comparative analysis between Figure 8a,b indicated that the design of ASLC‐B enhanced the material advantages of AlSi12Fe2.5Ni3Mn4, leading to a greater increase in ultimate load compared to the improvement seen in PLC over PLC(AlSi10Mg) under identical conditions.

Figure 8c illustrated that ASLC‐B(AlSi10Mg) exhibited a superior energy absorption capacity compared to ASLC‐B at 25 °C, while its MCF was lower. This was likely that ASLC‐B tended to go through instantaneous fracture at room temperature, resulting in decreased energy absorption. However, the reduced failure displacement in ASLC‐B resulted in higher MCF and CFE. At 100 and 200 °C, ASLC‐B and ASLC‐B(AlSi10Mg) exhibited similar CFE values, indicating comparable energy absorption efficiency between the 2 lattice cell structures. However, ASLC‐B maintained higher SEA and MCF than ASLC‐B(AlSi10Mg) thanks to its enhanced load‐bearing capacity and absence of structural instantaneous fracture at high temperatures.

Figure 9b showed that micropores were presented on the surfaces of both materials, and the fracture surfaces both exhibited ductile fracture characteristics. However, the fracture surface of the AlSi12Fe2.5Ni3Mn4 lattice structure was smoother than that of AlSi10Mg. Specifically, the fracture surface of ASLC‐B(AlSi10Mg) predominantly featured large and deep dimples, with dimple sizes ranging from 0.24 to 2.46 µm, showing well‐developed craters and holes. In contrast, the fracture surface of ASLC‐B mainly showed small and shallow dimples. Then, the comparison of the dimples indicated that the structure prepared using AlSi10Mg possessed better ductility. From 25 to 200 °C, both materials of the lattice structures exhibited similar ductile fracture modes. Observations indicated that as the temperature increased, the dimples on the fracture surface of ASLC‐B became larger and deeper, suggesting the improved ductility of AlSi12Fe2.5Ni3Mn4. However, the material strength decreased, aligning well with the trends observed in the load‐displacement curves obtained from experiments. In addition, as shown in Figure 9b, precipitated second‐phase particles were observed within the dimples on the fracture surface of ASLC‐B. These precipitated phases acted as initiation pores of dimples, facilitating the growth and linkage of microcracks. Extended microcracks appeared on the fracture surface of ASLC‐B at 25 and 100 °C, which continued to aggregate and expand across the entire surface, leading to sudden fracture of the overall structure. As the temperature increased, these microcracks within the dimples ceased to further propagate along the fracture surface, which explained why the sudden fracture was not observed in the test conducted at 200 °C.

Based on the EBSD results in Figure S1d,e (Supporting Information), AlSi12Fe2.5Ni3Mn4 exhibited a typical grain structure in the direction of the building surface, showing a mixture of columnar grains, coarse grains, fine grains, and columnar grains. A large number of nanocrystalline grains could be identified at the melt pool boundaries, with sizes ranging from 0.5 to 1 µm. In the center of the melt pool, columnar grains with lengths of 10 to 50 µm and widths of 2–5 µm were distributed. Statistical results showed that the average grain size is 2.74 µm, indicating a significant grain refinement effect in the alloy.

### 3.5 Effect on Heat Transfer

**Figure 10:**
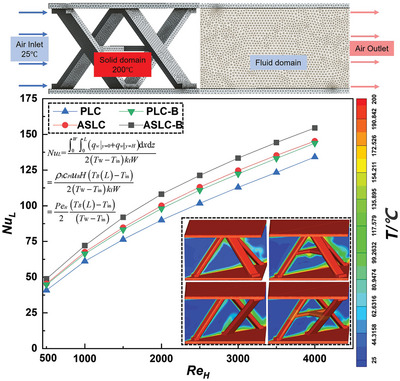
*Nu_L_*‐*Re_H_* curves and temperature clouds of PLC, PLC‐B, ASLC, and ASLC‐B.

According to the reference,^[^ ^57^, ^58^ ^]^ where *H* = 0.016 m, *Pr* = 0.71, *v_f_* = 1.58 × 10^−5^ m^2^/s, *k_f_* = 0.0242 W (m·K). The explanation of the parameters was in Section 5. As shown in **Figure** **10**, the model was divided into a fluid domain and a solid domain. Fluent Meshing was utilized to mesh the model. The first layer of the boundary layer was set at a thickness of 0.01 mm, consisting of a total of 5 layers. The flow and heat transfer simulations were conducted using Fluent. The initial structural temperature was set to 200 °C. Air at 25 °C was passed through the inlet at varying velocities: 0.403, 0.806, 1.209, 1.612, 2.015, 2.418, 2.821, 3.224 m s^−1^, corresponding to a range of *Re_H_* from 500 to 4000. By measuring *T_B_(x)* and *T_W_*, the relationship between *Nu_L_* and *Re_H_* could be obtained.

The *Nu_L_* of 4 lattice cells increased with the elevating of *Re_H_*, and the convective heat transfer capacity of the structure was enhanced. When *Re_H_* was 1500 (inlet flow velocity was 1.209 m s^−1^), ASLC‐B had the largest *Nu_L_*, which was 20.27% higher than PLC.

Under the same *Re_H_*, the *Nu_L_* of ASLC series consistently exceeded that of PLC series, demonstrating that the antisymmetric design effectively improved the heat transfer characteristics. Comparative analysis of the temperature clouds of PLC and ASLC revealed that ASLC generated larger temperature gradients. Despite having similar surface areas—9.96 cm^2^ for PLC and 10.01cm^2^ for ASLC, the difference in *Nu_L_* was substantial. This discrepancy was attributed to the antisymmetric design, which guaranteed more pronounced airflow disturbance over the same surface area, thereby facilitating more effective temperature exchange. As a result, the heat transfer capacity of antisymmetric lattice cells surpassed that of pyramid lattice cells.

Figure 10 showed that the *Nu_L_* values of PLC‐B and ASLC‐B with the cross‐core design were higher than those of their counterparts without cross‐cores, indicating that the introduction of a cross‐core successfully boosted the heat transfer performance of the structure. At the same *Re_H_*, the *Nu_L_* of PLC‐B increased by 8.46% over PLC, and the *Nu_L_* of ASLC‐B increased by 9.05% over ASLC. The temperature clouds revealed a pronounced temperature gradient at the cross‐core of ASLC‐B attributed to its largest surface area of 12.25 cm^2^, being 22.78% larger than ASLC, resulting in the most extensive contact area with the airflow and thereby increasing heat transfer efficiency. The presence of cross‐core also affects the airflow dynamics by causing obstruction and drag effects that generate turbulence on both sides of the core, enhancing the convective effect and accelerating heat dissipation. Structures featuring the cross‐core formed a flow band of a certain width and higher temperature, facilitating sufficient temperature exchange and the generation of temperature gradients that enhanced the convective heat transfer. As the airflow passed through the cross‐core, it encountered a large temperature gradient, which further intensified the heat transfer process. Therefore, the cross‐core not only increased the surface area but also significantly influenced airflow behavior, leading to improved heat transfer performance of the structure.

## 4 Conclusion

This study presented a comprehensive exploration of the configuration design, preparation, characterization, performance testing, and comparative analysis of the newly designed AlSi12Fe2.5Ni3Mn4 antisymmetric lattice cell, employing both experimental and numerical approaches. The specific conclusions obtained were as follows:

1) At temperatures of 25, 100, and 200 °C, the ultimate load of ASLC‐B increased by 36.04%, 37.17%, and 38.85% respectively, compared to PLC. Additionally, the heat transfer efficiency increased by 27.34%. Moreover, the high‐precision numerical model based on J‐C constitutive demonstrated that ASLC‐B optimized stress distribution, effectively reducing structural buckling and delaying the onset of failure.
2) With rising temperatures (100 and 200 °C), although the ultimate load of ASLC‐B decreased by 7.74% and 28.13%, and the structural stress by 4.07% and 14.05%, respectively, the specific energy absorption (SEA) increased by 58.77% and 41.23%. In addition, the failure mode shifted from an instantaneous fracture of the core at room temperature to buckling of the core at a higher temperature.
3) When comparing different materials, ASLC‐B exhibited a 39.39% (25 °C), 51.56% (100 °C), and 44.14% (200 °C) higher load‐bearing capacity than ASLC‐B(AlSi10Mg). Scanning electron microscopy (SEM) showed that while lattice structures made from both materials exhibited ductile fracture modes at different temperatures, AlSi12Fe2.5Ni3Mn4 achieved higher strength and slightly reduced ductility due to the addition of elements resistant to high‐temperature creep.

## 5 Experimental Section

### Methods of Testing

**Figure 11:**
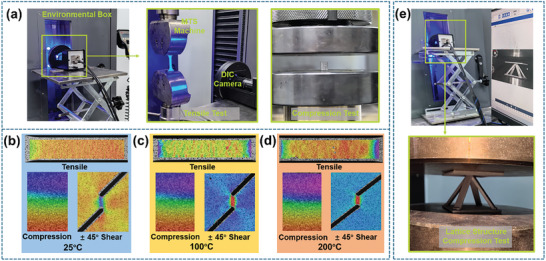
Experimental schematics: a) schematic of basic mechanical performance test devices; b–d) DIC schematics of basic mechanical performance tests at 25, 100, and 200 °C; e) schematic of lattice structure quasi‐static compression test device.

To obtain the basic mechanical performance parameters of the AlSi12Fe2.5Ni3Mn4 aluminum alloy, tensile and compression tests were conducted according to ASTM E8/E8M^[^ ^59^ ^]^ and ASTM E9.^[^ ^60^ ^]^ Additionally, shear tests and thermal expansion coefficient measurements were conducted according to ASTM B831^[^ ^61^ ^]^ and ASTM C531.^[^ ^62^ ^]^ The tests were carried out at different environmental temperatures (25, 100, 200 °C) with a reference strain rate of 0.001 s^−1^, and each test group contained 5 specimens. During the tests, a combination of static strain testing‐analysis system and digital image correlation (DIC) was employed to collect strain data within the test area. The testing setup and the DIC device are shown in **Figure** **11**a, and the schematics of DIC strain results (25, 100, 200 °C) are shown in Figure 11b–d, respectively.

To investigate the load‐bearing capabilities of the antisymmetric lattice structure fabricated from the newly developed AlSi12Fe2.5Ni3Mn4 at various temperatures, this section described tests on 4 configurations of lattice cells (PLC, PLC‐B, ASLC, ASLC‐B) across 12 groups of quasi‐static compression tests at environmental temperatures of 25, 100, and 200 °C, with 5 test pieces in each group. For comparative analysis, identical tests were also conducted on the same 4 lattice structures fabricated from the conventional AlSi10Mg.

According to the ASTM C365/C365M‐22,^[^ ^63^ ^]^ all lattice cell structures were produced using SLM technology. The compression test setup and the actual loading conditions are illustrated in Figure 11e. The tests were conducted using an MTS‐E42 Universal Testing Machine equipped with a 500 °C Environmental Box at a loading rate of 1 mm min^−1^.

### Numerical Methods of Thermal‐mechanical Coupling Testing

During the initial linear elastic phase, the classical elastic stress‐strain relationship was employed. However, once the material surpassed its initial yield stress and transitioned into the plastic phase, the Johnson‐Cook (J‐C) constitutive model was utilized to define the constitutive relationship.

The constitutive relationship in the elastic phase mainly adhered to the generalized Hooke's law, as shown in Equations (5)–(7):

(5) $$\varepsilon_{x}=\frac{1}{E}\;\left[\sigma_{x}-\mu \left(\sigma_{y}+\sigma_{z}\right)\right],\;\gamma_{\mathrm{yz}}=\frac{\tau_{\mathrm{yz}}}{2G}$$

(6) $$\varepsilon_{y}=\frac{1}{E}\;\left[\sigma_{y}-\mu \left(\sigma_{x}+\sigma_{z}\right)\right],\;\gamma_{\mathrm{xz}}=\frac{\tau_{\mathrm{xz}}}{2G}$$

(7) $$\varepsilon_{z}=\frac{1}{E}\;\left[\sigma_{z}-\mu \left(\sigma_{x}+\sigma_{y}\right)\right],\;\gamma_{\mathrm{xy}}=\frac{\tau_{\mathrm{xy}}}{2G}$$

In the above equations, *ε_i_* and *σ_i_* are the normal strain and normal stress; *γ_ij_* and *τ_ij_* are the shear strain and shear stress (*i*, *j* = *x*, *y*, *z*); *E* is the Elastic modulus; *G* is the Shear modulus; *µ* is the Poisson's ratio. According to the classical elastoplastic constitutive model, accurately input the material parameters *E* and *µ* during the elastic phase in ABAQUS.

The J‐C constitutive model took into account the coupling effects of deformation, strain rate, and environmental temperature in the plastic phase of metal materials. This comprehensive approach enabled the J‐C constitutive model to better characterize the plastic deformation phase of materials across different temperature environments, offering a distinct advantage over the classical elastoplastic constitutive model.^[^ ^64^, ^65^ ^]^ The specific form of the model is as follows:

(8) $$\sigma =\left[A+B\varepsilon_{e}^{n}\right]\left[1+\mathrm{Cln}\frac{\varepsilon_{e}^{\cdot }}{\varepsilon_{0}^{\cdot }}\right]\left[1-{\left(\frac{T-T_{r}}{T_{m}-T_{r}}\right)}^{m}\right]$$

In the above equations, *A* is the initial yield stress at the reference strain rate and temperature; *B* is the strain hardening modulus; *n* is the hardening exponent; *C* is the strain rate strengthening coefficient; *m* is the temperature softening exponent; $\varepsilon_{0}^{.}$ is the reference strain rate; *ε_e_* is the equivalent plastic strain; $\varepsilon_{e}^{.}$ is the equivalent plastic strain rate; *T*, *T_r_*, and *T_m_* are the environmental temperature, reference temperature, and melting temperature, respectively.

Specific test methods and operating procedures for determining J‐C constitutive parameters in quasi‐static tension were described in Section 5.1.

Based on the test results detailed in Section 2.2, with ε^·^  = 0.001 s^−1^ and *T *= 25 °C, under quasi‐static conditions at room temperature, the AlSi12Fe2.5Ni3Mn4 can be approximated as a linearly strengthened elastoplastic material, and the J‐C constitutive equation can be consequently simplified to Equation (9). The initial yield stress of the material under room temperature quasi‐static conditions can be obtained from the tensile curve, corresponding to the value of *A*. Through least squares fitting according to Equation (10), *B* and *n* can be obtained.

(9) $$\sigma =A+B\varepsilon_{e}^{n}$$

(10) $$\ln\left(\sigma -A\right)=\ln B+n\ln\varepsilon_{e}$$

Supplement the tensile tests at ε^·^ = 0.001s^−1^, *T* = 150 °C and 300 °C, then calculate *m*; supplement the tensile tests at *T *= 25 °C, ε^·^ = 0.1, 0.01, and 0.0001 s^−1^, then calculate *C*. There were 5 groups of supplementary tests, with 5 specimens in each group.

Under quasi‐static conditions with a specified strain ε = 0.1, the J‐C constitutive equation can be simplified as:

(11) $$\sigma_{0.1}=\sigma_{0}\left[1-{\left(\frac{T-T_{r}}{T_{m}-T_{r}}\right)}^{m}\right]$$

(12) $${\left(\frac{T-T_{r}}{T_{m}-T_{r}}\right)}^{m}=1-\frac{\sigma_{0.1}}{\sigma_{0}}$$

(13) $$\ln\left(1-\frac{\sigma_{0.1}}{\sigma_{0}}\right)=\mathrm{mln}\left(\frac{T-T_{r}}{T_{m}-T_{r}}\right)$$

In the above equations, *σ_0.1_* is the tensile yield stress of AlSi12Fe2.5Ni3Mn4 when the strain is 0.1 at different temperatures, *σ_0_* is the tensile yield stress when the strain is 0.1 at 25 °C, and *m* can be obtained by least squares fitting according to Equation (13).

At *T* = 25 °C, under different strain rates, the J‐C constitutive equation can be simplified as:

(14) $$\sigma =\left[1+\frac{C\ln\varepsilon_{e}^{\cdot }}{\varepsilon_{0}^{\cdot }}\right]$$

(15) $$\frac{\sigma_{i}}{\sigma_{0}}-1=\frac{C\ln\varepsilon_{e}^{\cdot }}{\varepsilon_{0}^{\cdot }}$$

In the above equations, *σ_0_* is the tensile yield stress at a strain rate of 0.001 s^−1^, taking the strain ε_0_ at this strain rate as the reference strain; *σ_i_* is the tensile yield stress at different strain rates; and *C* can be obtained by least squares fitting according to Equation (15).

Figure 3a displays the finite element model of ASLC‐B established in ABAQUS, consisting of an upper panel, lower panel, and cores. Material properties for the entire model were determined based on the results of the basic mechanical performance tests and the J‐C constitutive parameters. Although SLM printing technology may impart a certain degree of anisotropy to the material, for computational feasibility and accuracy, the material was approximated as isotropic in the numerical simulations, being a common simplification in such analyses.^[^ ^66^, ^67^ ^]^ To accurately simulate different temperature environments, a predefined temperature field was integrated into the overall structure. This enabled thermal‐mechanical coupling simulations of lattice structures at different temperatures. In the simulation setup, the lower panel was fixed, and a vertical displacement load was applied to the upper panel, coupled to the reference point RP‐1.

### Numerical Methods of Heat Transfer

The Nusselt number (*Nu*) is a dimensionless number that qualifies the ratio of convective heat transfer to thermal conduction in a fluid. It is a commonly used metric in describing the convective heat transfer capability of a fluid and serves as one of the key parameters for evaluating the heat transfer performance of structures within engineering applications. The Nusselt number based on the characteristic length of the flow direction of a pipe (*Nu_L_*) was defined following the principle of energy conservation, as shown in Figure 10, where the Peclet number (*Pe_H_*) characterizes the ratio of convective rate to diffusive rate, and is defined as:

(16) $$Pe_{H}=\frac{\rho_{f}c_{\mathrm{pf}}u_{B}H}{k_{f}}=Re_{H}\;\mathrm{Pr}$$

Prandtl number (*Pr*) characterizes the relative size of the flow boundary layer and the thermal boundary layer, which is defined as:

(17) $$\mathrm{Pr}=\frac{v_{f}}{\alpha_{f}}\;$$

Reynolds number (*Re_H_*) is defined as:

(18) $$Re_{H}=\frac{u_{B}H}{v_{f}}\;$$

*v_f_* is the kinematic viscosity coefficient; *k_f_* is air thermal conductivity; *H* is the height of the lattice cell; *T_B_(x)* is the cross‐sectional temperature at the outlet; *T_W_* is the wall temperature; *u_B_* is the average velocity of the channel cross‐section.

## Conflict of Interest

The authors declare no conflict of interest.

## Supporting information

Supporting Information

## Data Availability

The data that support the findings of this study are available from the corresponding author upon reasonable request.
