# Supplementary material for: Thermal‐Mechanical Coupling Performance of Heat‐Resistant, High‐Strength and Printable Al‐Si Alloy Antisymmetric Lattice Structure
Source: Adv Sci (Weinh). 2024 Aug 29;11(42):2407107. doi: 10.1002/advs.202407107 (PMC11558119; doi:10.1002/advs.202407107)
Supplement: Supplementary file 1 — Supporting Information [file ADVS-11-2407107-s001.docx]

Supporting Information

Thermal-mechanical Coupling Performance of Heat-resistant, High-strength and Printable Al-Si Alloy Antisymmetric Lattice Structure

Jiaqi Yan^§^, Zhicheng Dong^§^, Ben Jia^§^, Shunshun Zhu, Guowei Li, Yuhao Zheng, Heyuan Huang*

^§^Contributed equally to this work

J. Q. Yan, Y. H. Zheng, H. Y. Huang

School of Aeronautics

Northwestern Polytechnical University

Xi’an 710072, China

E-mail: huangheyuan@nwpu.edu.cn

Z. C. Dong, B. Jia, S. S. Zhu, G. W. Li

School of Civilaviation

Northwestern Polytechnical University

Xi’an 710072, China

H. Y. Huang

National Key Laboratory of Aircraft Configuration Design

Xi’an 710072, China

**
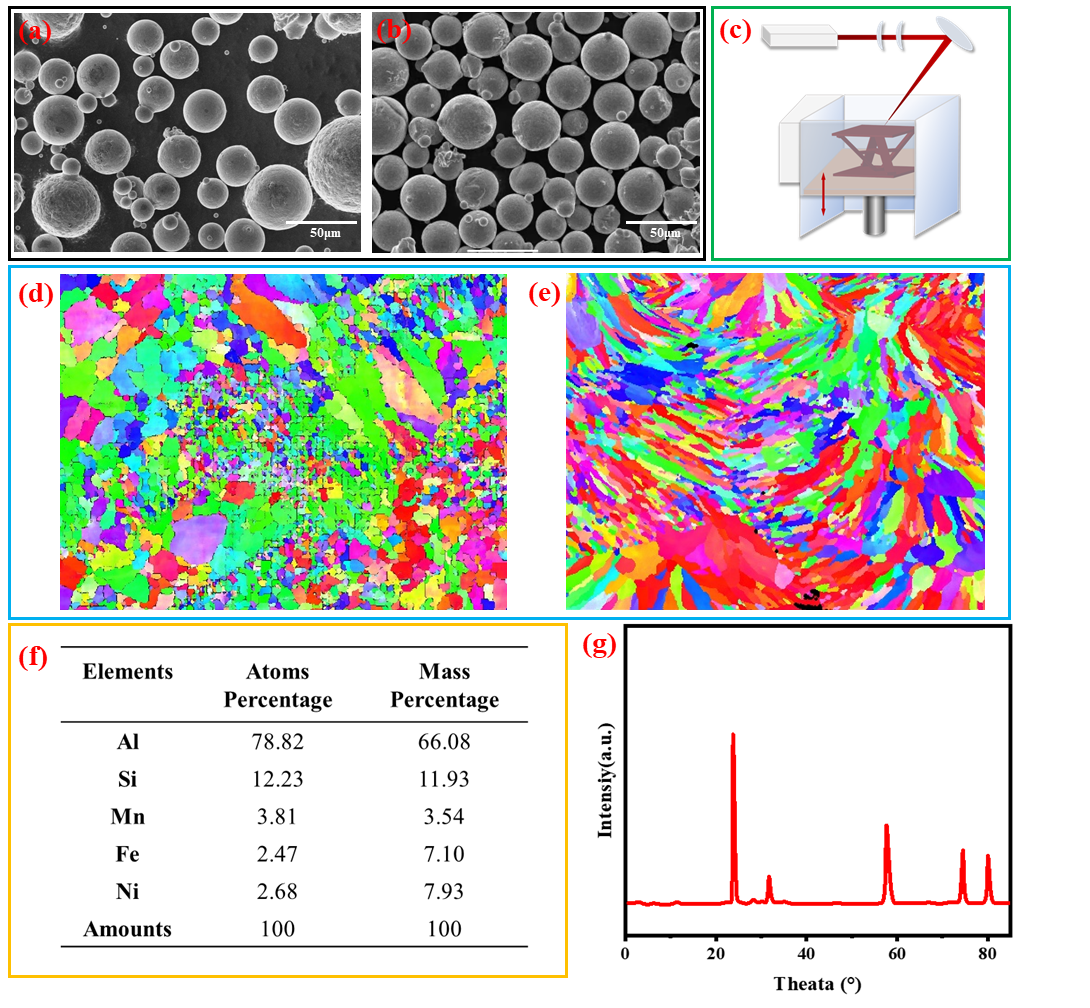
**

**Figure S1.** (a) SEM image of AlSi12Fe2.5Ni3Mn4 powder; (b) SEM image of AlSi10Mg powder; (c) SLM production process diagram; EBSD orientation map: (d) horizontal cross-sections, (e)vertical cross-sections; (f) EDS analysis result of SLM AlSi12Fe2.5Ni3Mn4 alloy samples; (g) XRD analysis result of SLM AlSi12Fe2.5Ni3Mn4 alloy samples.

**
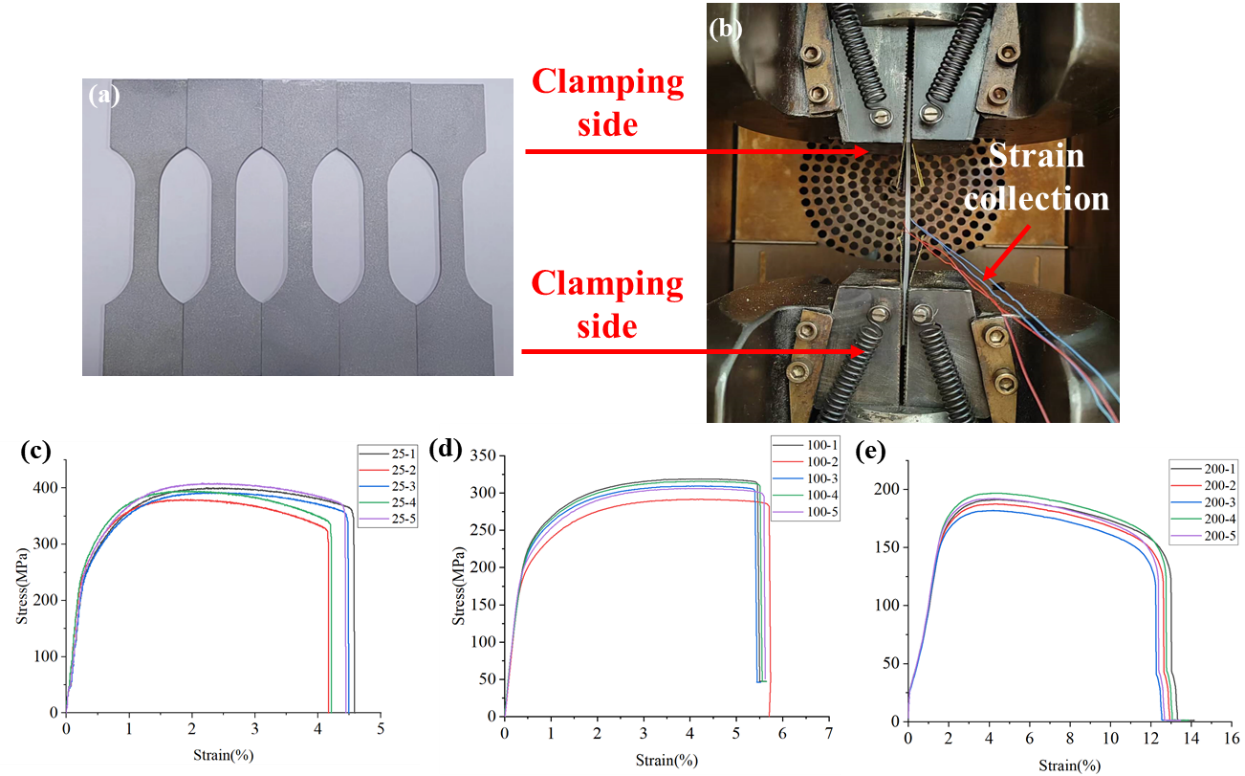
**

**Figure S2.** (a) (a) AlSi10Mg tensile test specimen; (b) Schematic diagram of tensile test; Stress-strain curves for tensile testing at (c)25℃, (d)100℃, and (e)200℃.
